# Correlation between remnant cholesterol and premature coronary artery disease and the severity of coronary artery lesions in men: a retrospective study

**DOI:** 10.3389/fcvm.2024.1462142

**Published:** 2024-11-25

**Authors:** Xingming Dong, Ke Chen, Xiuqin Li, Yuanyuan Tang, Rui Zhang, Jian Wang

**Affiliations:** ^1^ Department of Cardiology, The First Affiliated Hospital of Shandong Second Medical University, Weifang People’s Hospital, Weifang, Shandong, China; ^2^ Key Laboratory of Cardiopulmonary-Cerebral Resuscitation Research of Weifang, The First Affiliated Hospital of Shandong Second Medical University, Weifang People’s Hospital, Weifang, Shandong, China; ^3^ Clinical Laboratory, Weifang Second People’s Hospital, Weifang, Shandong, China; ^4^ Translational Medical Center, Weifang Second People’s Hospital, Weifang, Shandong, China

**Keywords:** premature coronary artery disease, remnant cholesterol, coronary artery lesions, Gensini score, retrospective study

## Abstract

**Objective:**

To investigate the correlation between remnant cholesterol (RC) and premature coronary artery disease (PCAD) and the severity of coronary artery lesions in men.

**Methods:**

A total of 630 male subjects who underwent coronary angiography were included in the study. The general data, laboratory tests, and coronary angiography data of each group were statistically analyzed, and RC levels were calculated. According to the Gensini score, PCAD was divided into mild and severe lesion groups. The relationship between RC and PCAD and coronary artery lesions was analyzed using multivariate logistic regression and spearman correlation analysis, and the predictive value of RC for coronary artery lesions was evaluated using receiver operating characteristic (ROC) curves.

**Results:**

The RC levels in the PCAD group were significantly higher than those in the non-PCAD group (*p* < 0.05), and RC was an independent risk factor for PCAD (*p* < 0.05).The RC levels in the severe lesion group were higher than those in the mild lesion group (*p* < 0.05), and RC levels were positively correlated with the Gensini score (*r* = 0.335, *p* < 0.001).Multivariate logistic regression analysis showed that RC was an independent risk factor for severe coronary artery lesions (*p* < 0.05).The ROC curve calculated the value of RC in predicting severe coronary artery lesions, with an area under the curve of 0.693, a cutoff value of 0.485 mmol/L, a sensitivity of 64.7%, and a specificity of 66.2%.

**Conclusion:**

RC is an independent risk factor for PCAD and the severity of coronary artery lesions in adult men. RC levels are positively correlated with the severity of coronary artery lesions and can be used as an auxiliary indicator for clinical assessment of PCAD.

## 1 Introduction

In recent years, economic development and changes in lifestyle and dietary habits have made coronary artery disease (CAD) a leading cause of cardiovascular-related deaths. Notably, CAD is increasingly affecting younger populations. In patients with CAD, 4%–10% of patients with acute myocardial infarction (AMI) occur before the age of 45 (1), and among individuals aged 35 to 44, 62% of causes for sudden cardiac death can be attributed to CAD (2). PCAD is defined as CAD occurring in males under 55 years and females under 65 years (3). Although most studies indicate a low incidence rate for PCAD, the potential number of affected individuals may be significantly higher than currently estimated due to the rise in cardiovascular risk factors among younger populations. Statistics show that over half of PCAD patients experience significant coronary atherosclerosis progression within 10 years, with approximately 20% dying prematurely (4). Despite current preventive measures, PCAD still exhibits a high recurrence rate and mortality, intensifying the medical burden on younger populations and society (5). Therefore, it is crucial to identify high-risk PCAD populations early, provide interventions, and offer health guidance to reduce the incidence of PCAD.

Several major risk factors for CAD have been identified, including age, gender, smoking history, hypertension, diabetes, hyperuricemia, hyperlipidemia, obesity, and anxiety (6, 7). Among these, dyslipidemia is a significant independent risk factor in the occurrence and development of CAD. Traditionally, lipid-lowering therapy has focused on reducing low-density lipoprotein cholesterol (LDL-C). Research has shown that lowering LDL-C levels effectively improves CAD prognosis. However, many patients continue to experience major adverse cardiovascular events (MACEs), despite achieving target LDL-C levels and controlling other risk factors. Consequently, RC has emerged as a key marker of residual cardiovascular risk. RC is the cholesterol content in triglyceride-rich lipoproteins, including very low-density lipoprotein and intermediate-density lipoprotein in fasting states, and chylomicron remnants in non-fasting states (8). RC is now recognized as a residual cardiovascular risk factor even after achieving lipid targets, with substantial evidence linking it to CAD occurrence and progression (9).

Significant gender differences exist in CAD incidence, with men developing the disease at younger ages compared to women. Despite this, few studies have examined the correlation between RC and PCAD or the severity of coronary artery lesions in adult men. This study uses the Gensini score as the standard for evaluating the severity of coronary artery lesions to explore the correlation between RC and PCAD, and the severity of coronary artery lesions in adult men. The goal is to provide new insights into the prevention and treatment of PCAD in men.

## 2 Materials and methods

### 2.1 Study design and participants

**Figure 1:**
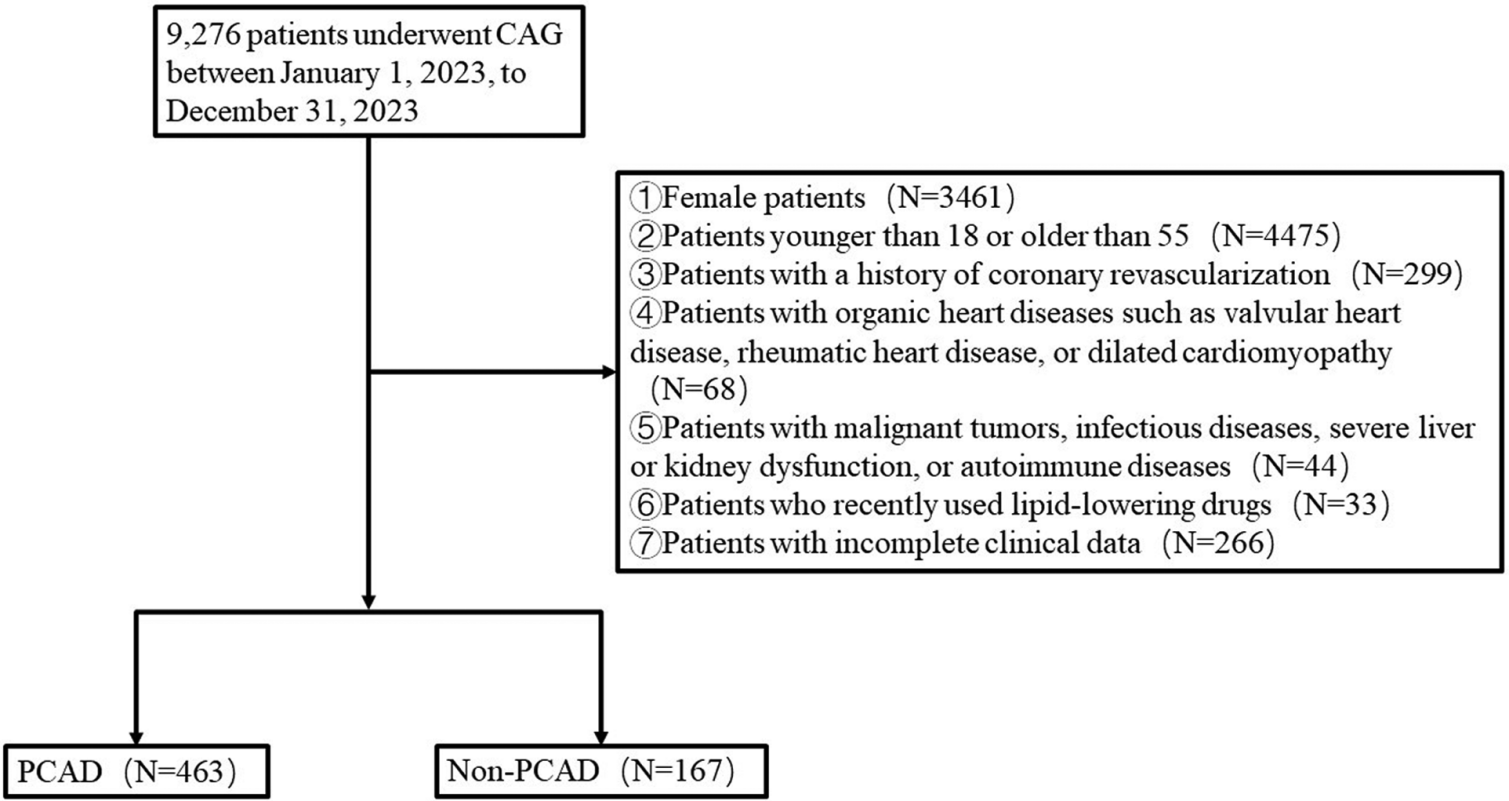
Flow chart.

From January 1, 2023, to December 31, 2023, a total of 9,276 patients suspected of having CAD and admitted to Weifang People's Hospital for coronary angiography were selected. The exclusion criteria were as follows: female patients; patients younger than 18 or older than 55; patients with a history of coronary revascularization; patients with organic heart diseases such as valvular heart disease, rheumatic heart disease, or dilated cardiomyopathy; patients with malignant tumors, infectious diseases, severe liver or kidney dysfunction, or autoimmune diseases; patients who recently used lipid-lowering drugs; patients with incomplete clinical data. Ultimately, 630 patients were included in the study, comprising 463 patients with PCAD and 167 patients without PCAD (Figure 1).

### 2.2 Data collection

Clinical data were collected from the Hospital Information System, including demographic characteristics, clinical history, laboratory results, and coronary angiography (CAG) findings. The demographic characteristics included age, height, weight, smoking status, drinking habits. Clinical history included the history of hypertension, diabetes and family history of CAD.

All blood samples were fasting venous samples collected within 24 h of admission. The laboratory indicators primarily included total cholesterol (TC), triglyceride (TG), high-density lipoprotein cholesterol (HDL-C), LDL-C, alanine aminotransferase (ALT), albumin (ALB), fasting blood glucose (FBG), uric acid (UA), and creatinine (CREA).

Invasive CAG was performed via percutaneous radial or femoral artery angiography. The angiographic equipment used was capable of accurately diagnosing all manifestations of coronary artery conditions.

### 2.3 Definitions

PCAD: (1) Age <55 years. (2) Obstructive stenosis ≥50% in the lumen diameter of any major coronary artery (including the left main coronary artery, left anterior descending artery, left circumflex artery, and right coronary artery) or their main branches.

BMI = Weight (kg)/Height (m)^2^

RC = TC (mmol/L)—HDL-C (mmol/L)—LDL-C (mmol/L) (10)

Gensini Score: The total score is calculated based on the Gensini scoring guidelines, which assess the severity of PCAD (11). The scoring is performed by experienced cardiologists. According to the median Gensini score, a score of <44 is defined as mild PCAD, and a score of ≥44 is defined as severe PCAD.

### 2.4 Statistical analysis

Statistical analysis was performed using SPSS 25.0. The normality of continuous variables was tested using the Kolmogorov-Smirnov (K-S) test. Non-parametric tests were used for comparisons if the variables were not normally distributed. Categorical variables were expressed as rates and compared using the chi-square test. The correlation between RC and the Gensini score was analyzed using Spearman's correlation. Continuous variables were converted to binary variables for multivariate logistic regression analysis to compare the relationship between laboratory indicators, risk factors, and the severity of coronary artery lesions. ROC curves were plotted, and the optimal cutoff value of RC for predicting severe coronary artery lesions was determined based on the Youden index. A *p*-value of less than 0.05 was considered statistically significant.

## 3 Results

### 3.1 Clinical and demographic characteristics of PCAD and non-PCAD groups

The clinical data analysis involved 630 subjects, including 463 newly diagnosed PCAD patients and 167 non-PCAD individuals (Table 1). The results showed that compared with the non-PCAD group, the PCAD group had a higher proportion of smokers and higher rates of hypertension, diabetes. ALT, FBG, TC, TG, LDL-C, and RC levels were significantly higher in the PCAD group than in the non-PCAD group, with differences being statistically significant (all *p* < 0.05). HDL-C was significantly lower in the PCAD group compared to the non-PCAD group, with a statistically significant difference (*p* < 0.05). There were no statistically significant differences between the two groups in terms of age, alcoholism, family history of CAD, BMI, ALB, UA,and CERA (all *p* > 0.05).

**Table 1:** Clinical and demographic characteristics of PCAD and non-PCAD groups.

Variables	PCAD (*n* = 463)	Non-PCAD (*n* = 167)	Z/*χ*^2^	*p*-value
Age (years)	49 (44, 52)	49 (45, 52)	−0.368	0.713
Hypertension, *n* (%)	218 (47.1%)	54 (32.3%)	10.882	0.001
Diabetes, *n* (%)	114 (24.6%)	15 (9%)	18.437	<0.001
Smoking, *n* (%)	275 (59.4%)	70 (41.9%)	15.136	<0.001
Alcoholism, *n* (%)	93 (20.1%)	26 (15.6%)	1.635	0.201
Family history of CAD, *n* (%)	62 (13.4%)	13 (7.8%)	3.678	0.055
BMI (Kg/m^2^)	26.99 (24.61, 28.73)	26.23 (23.88, 28.70)	−1.617	0.106
ALT (U/L)	28 (20, 38)	23 (16, 33)	−3.677	<0.001
ALB (g/L)	44 (41.3, 46.4)	44.3 (42.4, 46.9)	−1.499	0.134
FBG (mmol/L)	5.6 (5, 6.8)	5.1 (4.8, 5.7)	−5.948	<0.001
UA (umol/L)	359 (296, 424)	357 (303, 420)	−0.33	0.973
CERA (umol/L)	65 (56, 74)	66 (60, 72)	−1.063	0.288
TC (mmol/L)	4.7 (4.1, 5.5)	4.5 (3.8, 5.1)	−3.955	<0.001
TG (mmol/L)	1.74 (1.16, 2.66)	1.45 (1.05, 1.99)	−3.422	<0.001
HDL-C (mmol/L)	1 (0.88, 1.2)	1.17 (1.02, 1.33)	−6.759	<0.001
LDL-C (mmol/L)	3.19 (2.6, 3.83)	2.98 (2.36, 3.44)	−3.478	0.001
RC (mmol/L)	0.48 (0.31, 0.67)	0.28 (0.18, 0.44)	−9.372	<0.001

BMI, the body mass index; ALT, alanine aminotransferase; ALB, albumin; FBG, fasting blood glucose; UA, uric acid; CREA, creatinine; TC, total cholesterol; TG, triglycerides; HDL-C, high-density lipoprotein cholesterol; LDL-C, low-density lipoprotein cholesterol; RC, remnant cholesterol.

### 3.2 Relationship between RC and PCAD

Using the presence of PCAD as the dependent variable, a multivariate logistic regression analysis was conducted. The variables with *p* < 0.1 in univariate analysis were adjusted for. The results indicated that hypertension, smoking, and family history of CAD, ALT, FBG, LDL-C, and RC were independent risk factors for PCAD (all *p* < 0.05), while HDL-C was an independent protective factor against PCAD (*p* < 0.05) (Table 2).

**Table 2:** Multivariate logistic regression analysis of PCAD risk factors.

Variables	B	Wald	OR	95%CI	*p*-value
Hypertension, *n* (%)	0.451	4.053	1.571	1.012–2.437	0.044
Diabetes, *n* (%)	0.410	1.327	1.507	0.750–3.030	0.249
Smoking, *n* (%)	0.627	8.478	1.872	1.227–2.854	0.004
Family history of CAD, *n* (%)	0.891	5.892	2.439	1.187–5.009	0.015
ALT (U/L)	0.017	4.331	1.017	1.111–1.033	0.037
FBG (mmol/L)	0.380	11.528	1.462	1.174–1.820	0.001
HDL-C (mmol/L)	−1.937	20.361	0.144	0.062–0.334	<0.001
LDL-C (mmol/L)	0.403	10.181	1.497	1.168–1.971	0.001
RC (mmol/L)	3.772	39.352	43.453	13.373–141.187	<0.001

ALT, alanine aminotransferase; FBG, fasting blood glucose; HDL-C, high-density lipoprotein cholesterol; LDL-C, low-density lipoprotein cholesterol; RC, remnant cholesterol.

### 3.3 Clinical and demographic characteristics of different coronary artery lesion groups

The severity of coronary artery lesions was assessed using the Gensini score, with scores <44 defined as mild PCAD and scores ≥44 defined as severe PCAD. The results showed that, compared to the mild PCAD group, the severe PCAD group had a higher proportion of patients with diabetes. FBG, TG, and RC levels were significantly higher in the severe PCAD group than in the mild PCAD group, with statistically significant differences (all *p* < 0.05) (Table 3). HDL-C levels were significantly lower in the severe PCAD group compared to the mild PCAD group (*p* < 0.05). There were no statistically significant differences between the two groups in terms of age, hypertension, smoking, alcoholism, family history of CAD, BMI, ALT, ALB, UA, UREA, TC, and LDL-C (all *p* > 0.05).

**Table 3:** Clinical and demographic characteristics of mild PCAD group and severe PCAD group.

Variables	Mild PCAD group (*n* = 231)	Severe PCAD group (*n* = 232)	Z/χ^2^	*p*-value
Age (years)	50 (45, 52)	49 (44, 52)	−1.741	0.082
Hypertension, *n* (%)	107 (46.3%)	111(47.8%)	0.108	0.742
Diabetes, *n* (%)	41 (17.7%)	73 (31.5%)	11.734	0.001
Smoking, *n* (%)	130 (56.3%)	145 (62.5%)	1.859	0.173
Alcoholism, *n* (%)	50 (21.6%)	43 (18.5%)	0.698	0.404
Family history of CAD, *n* (%)	33 (14.3%)	29 (12.5%)	0.318	0.573
BMI (Kg/m^2^)	26.75 (24.62, 28.41)	27.04 (24.52, 29.24)	−0.637	0.524
ALT(U/L)	27 (19, 37)	30 (21, 40.75)	−1.867	0.062
ALB (g/L)	43.9 (41.3, 46.1)	44.2 (41.4, 46.7)	−0.630	0.529
FBG (mmol/L)	5.5 (4.8, 6.2)	5.9 (5.2, 7.8)	−4.502	<0.001
UA (umol/L)	358 (306, 424)	360 (287, 424)	−0.916	0.360
CERA (umol/L)	66 (56, 75)	64 (56, 73)	−0.827	0.408
TC (mmol/L)	4.7 (4.1, 5.4)	4.8 (4.2, 5.6)	−0.986	0.324
TG (mmol/L)	1.60 (1.08, 2.29)	1.88 (1.23, 3.03)	−2.635	0.008
HDL-C (mmol/L)	1.07 (0.93, 1.24)	0.95 (0.82, 1.13)	−5.176	<0.001
LDL-C (mmol/L)	3.20 (2.622, 3.80)	3.18 (2.60, 3.90)	−0.157	0.876
RC (mmol/L)	0.38 (0.26, 0.53)	0.56 (0.4, 0.76)	−7.190	<0.001

BMI, the body mass index; ALT, alanine aminotransferase; ALB, albumin; FBG, fasting blood glucose; UA, uric acid; CREA, creatinine; TC, total cholesterol; TG, triglycerides; HDL-C, high-density lipoprotein cholesterol; LDL-C, low-density lipoprotein cholesterol; RC, remnant cholesterol.

### 3.4 Correlation analysis between the severity of coronary artery lesions and RC

Spearman correlation analysis showed a significant correlation between RC and the Gensini score (*r* = 0.335, *p* < 0.001). This indicates that as RC levels increase, the severity of coronary artery lesions in PCAD patients progressively worsens.

### 3.5 RC was an independent risk factor for severe coronary artery lesions

Using the presence of severe coronary artery lesions as the dependent variable, a multivariate logistic regression analysis was conducted. The variables with *p* < 0.1 in univariate analysis were adjusted for. The results indicated that FBG and RC were independent risk factors for severe coronary artery lesions (*p* < 0.05), while HDL-C was an independent protective factor against severe coronary artery lesions (*p* < 0.05) (Table 4).

**Table 4:** Multivariate logistic regression analysis of coronary artery lesions risk factors.

Variables	B	Wald	OR	95%CI	*p-value*
Hypertension, *n* (%)	−0.034	0.028	0.967	0.652–1.433	0.866
Diabetes, *n* (%)	0.404	2.141	1.498	0.872–2.576	0.143
Smoking, *n* (%)	0.244	1.468	1.276	0.860–1.893	0.226
ALT (U/L)	0.011	2.668	1.011	0.998–1.025	0.102
FBG (mmol/L)	0.105	4.166	1.111	1.004–1.230	0.041
HDL-C (mmol/L)	−1.681	16.235	0.186	0.082–0.422	<0.001
RC (mmol/L)	0.768	8.375	2.154	1.281–3.623	0.004

ALT, alanine aminotransferase; FBG, fasting blood glucose; HDL-C, high-density lipoprotein cholesterol; RC, remnant cholesterol.

### 3.6 Predictive value of RC for severe coronary artery lesions

**Figure 2:**
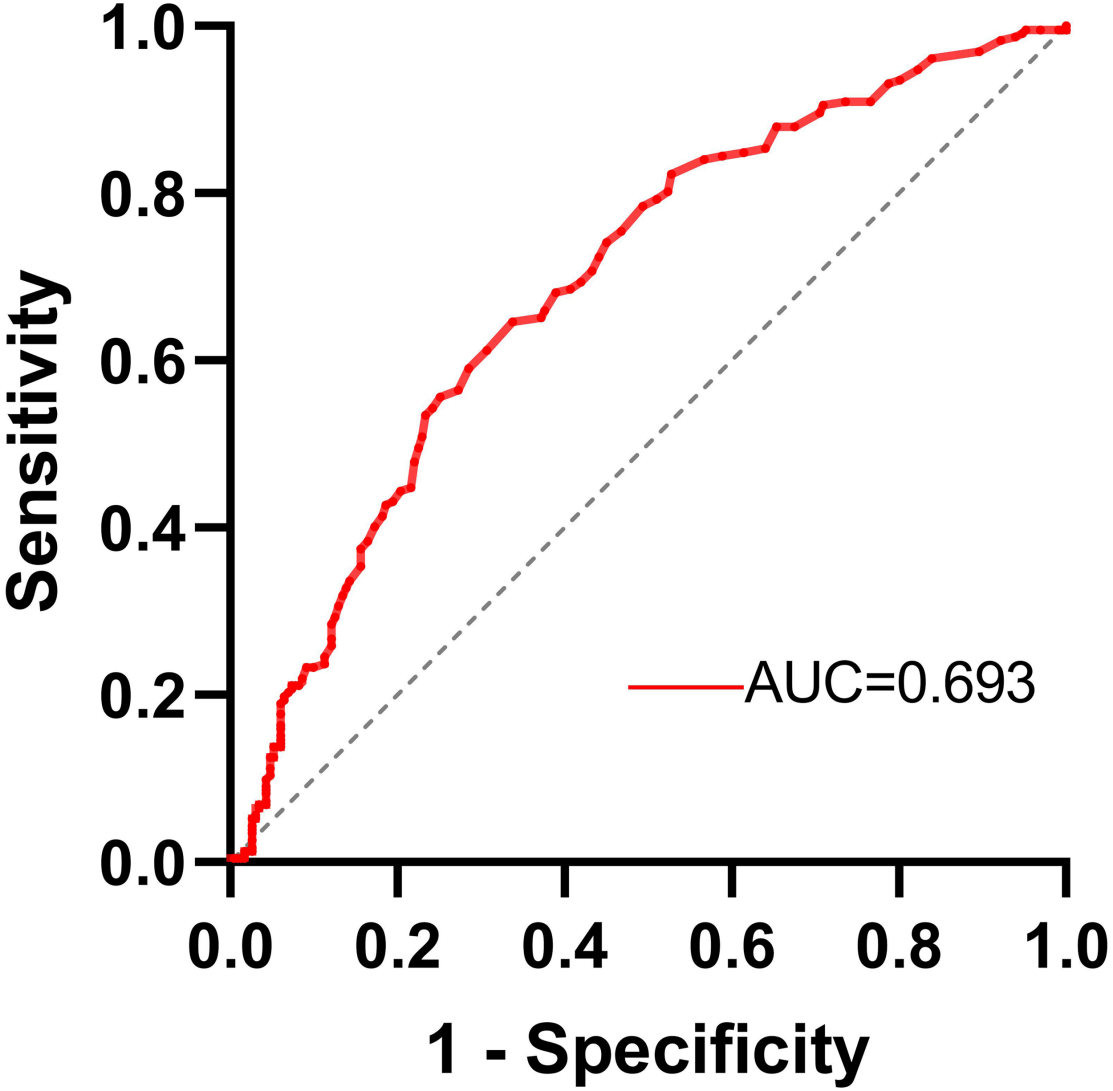
ROC curve analysis of RC for severe coronary artery lesions prediction.

The value of RC in predicting severe coronary artery lesions was assessed using ROC curve analysis (Figure 2). The results showed that the area under curve (AUC) was 0.693 (95% CI 0.645–0.741, *p* < 0.001). The optimal cutoff value for RC was determined to be 0.485 mmol/L, with a sensitivity of 64.7% and a specificity of 66.2%.

## 4 Discussion

PCAD is a special type of CAD characterized by early onset and rapid progression, leading to considerable psychological and physiological harm to patients. Hence, early identification of clinical risk factors, along with timely diagnosis and treatment, is crucial (12). In this retrospective study, we compared the clinical data of populations with PCAD and non-PCAD, and examined the correlation between RC and the severity of coronary artery lesions. Our results indicated that the RC levels in the PCAD group were significantly higher than those in the non-PCAD group. Notably, RC did not correlate with traditional cardiovascular risk factors. However, after adjusting for other variables, RC showed a positive correlation with the severity of coronary artery lesions. Therefore, RC demonstrates significant predictive value for the severity of CAD.

Lipid metabolism disorders are significant pathogenic factors in the progression of atherosclerosis, making active lipid control a crucial strategy in preventing and treating CAD. Our study identified lipid metabolism abnormalities in PCAD patients, consistent with the findings of previous research (13). Research indicates that a 1 mmol/L reduction in LDL-C lowers cardiovascular risk by 22%, and maintaining TG levels below 100 mg/dl reduces the likelihood of future MACEs by 50% (14, 15). However, many patients continue to experience recurrent cardiovascular events despite appropriate LDL-C and TG control. Recent genetic and epidemiological studies have increasingly confirmed the association between high RC levels and increased cardiovascular disease risk (16, 17). Fujihara et al. (18) conducted a 38-month follow-up of 247 patients with stable CAD who maintained LDL-C levels below 70 mg/dl, finding that 33 patients experienced cardiovascular events related to RC levels, and RC was identified as a significant predictor of major endpoints (hazard ratio 1.62, 95% confidence interval 1.26–2.07, *p* < 0.01). A study evaluating 1,716 patients with acute coronary syndrome (ACS) who underwent percutaneous coronary intervention (PCI) found that elevated RC levels (>0.79 mmol/L) were significantly associated with cardiovascular MACEs (19). Quispe et al. (20) analyzed 17,532 individuals without known CAD and found that elevated RC levels were associated with CAD, independent of traditional risk factors such as LDL-C and apolipoprotein B (apo B) levels. Luo et al. (21) discovered that fasting RC is an independent risk factor for in-stent restenosis (ISR) following PCI and a reliable predictor of ISR. In a prospective cohort study, elevated cumulative remnant cholesterol was significantly associated with the risk of developing ischemic heart disease (IHD), indicating consistent monitoring and modulation of RC may be instrumental in the prevention of IHD (22). These findings underscore the need for more comprehensive lipid management strategies that consider RC levels alongside traditional lipid parameters.

Our study found a positive correlation between the severity of coronary artery lesions and RC in PCAD patients, independent of LDL-C levels. This suggests that RC may influence the progression of cardiovascular disease through distinct pathways. Researchers have noted that elevated RC levels are associated with an increased risk of early atherosclerosis and contribute to the pathogenesis of CAD (23–25). Wu et al. (26) discovered that higher concentrations of RC are significantly correlated with the progression of atherosclerosis, and that screening for RC can help assess the extent of coronary atherosclerosis. Unlike LDL-C, RC can penetrate the vessel wall and is directly degraded by scavenger receptors in macrophages without undergoing oxidative modification (27). This leads to the formation of foam cells and promotes the development and progression of atherosclerotic plaques. Additionally, RC also participates in the formation and progression of atherosclerosis by activating monocyte-macrophages and upregulating pro-inflammatory cytokines (28, 29). In a study involving 60,608 patients with IHD, elevated non-fasting RC levels were associated with low-grade inflammation and IHD (30). Liao et al. (31) studied 5,778 CAD patients treated with statins and undergoing PCI, discovering that high residual inflammation and cholesterol risk, assessed by high-sensitivity C-reactive protein and RC were closely related to an increased risk of ischemic events, cardiovascular death, and all-cause mortality. Wu et al. (32) found that higher concentrations of RC are associated with an increased risk of hypertension and type 2 diabetes, potentially mediated by inflammatory responses involving leukocytes, lymphocytes, monocytes, and neutrophils. Given RC's unique role in atherogenesis and inflammation, it represents a potential target for more comprehensive lipid management strategies.

Our study also confirmed the impact of traditional risk factors such as hypertension, diabetes, smoking, and LDL-C on the development of PCAD, while HDL-C may be an independent protective factor against PCAD. In multivariate logistic regression analysis, blood glucose was closely related to the occurrence of PCAD and was an independent risk factor for the severity of coronary artery lesions, which may be associated with different states of glucose metabolism (33). The CAD group with concurrent diabetes represents a special cohort whose residual risk should receive more attention. Wang et al. (34) found that an elevated triglyceride-glucose (TyG) index, which reflects insulin resistance, was associated with an increased risk of multi-vessel CAD, and TyG may be a valuable predictor of CAD severity, particularly in individuals with prediabetes. Another follow-up study involving 4,095 diabetic patients found that RC levels were associated with all-cause and cardiovascular mortality, suggesting that maintaining appropriate RC levels may help reduce mortality risk in diabetic patients (35). In high cardiovascular risk diabetic patients, RC was closely associated with MACEs, independent of traditional risk factors and LDL-C, and its visit-to-visit variability was an independent predictor of adverse cardiovascular events (36). These findings highlight the need for a more comprehensive approach to managing lipid profiles in patients with CAD, particularly those with concurrent diabetes or prediabetes. In clinical practice, in addition to early detection and appropriate management of blood glucose and LDL-C, RC levels should also be actively monitored. Overall, RC can be widely used in clinical settings to identify high-risk individuals and may serve as a potential target for future therapeutic interventions.

The advantage of this study is that it focuses on adult males, a group with a high incidence of CAD, and explores the predictive value of RC for adult male PCAD. However, several limitations should be considered. Firstly, this study is a single-center retrospective study, which may introduce selection bias in the included population. Secondly, the sample size is relatively small, and larger sample sizes are needed to validate the results. Thirdly, we used estimated RC levels, which might not be as accurate as direct measurements, but this approach avoids additional costs in the study. Fourthly, this study did not include left ventricular ejection fraction (LVEF), and the interactive relationship between RC and LVEF with PCAD requires further investigation. Finally, this study did not include dynamic monitoring of RC levels in PCAD patients or follow-up of outpatient endpoint events.

## 5 Conclusions

RC is an independent risk factor for PCAD in adult men and is closely associated with the severity of coronary artery stenosis, demonstrating good predictive capability. Clinically, RC can be used as one of the parameters for dyslipidemia to optimize lipid-lowering regimens, facilitate early identification of high-risk individuals for CAD, and reduce the residual risk in CAD patients. This provides more accurate and effective monitoring and new prevention strategies for the clinical management of CAD.

## Data Availability

The raw data supporting the conclusions of this article will be made available by the authors, without undue reservation.
